# Reducing Sodium Content of Foods Served in Arkansas’s Largest School District: Evaluation of the Sodium Reduction in Communities Program

**DOI:** 10.5888/pcd19.220051

**Published:** 2022-09-01

**Authors:** Christopher R. Long, Brett Rowland, Matthew Gannon, Bonnie Faitak, Gena Smith, Jennifer Clampitt, Krista Langston, Jessica H. Presley, Emily S. English, Pearl A. McElfish

**Affiliations:** 1 College of Medicine, University of Arkansas for Medical Sciences Northwest, Fayetteville, Arkansas; 2 Office of Community Health and Research, University of Arkansas for Medical Sciences Northwest, Fayetteville, Arkansas; 3 Child Nutrition, Springdale Public Schools, Springdale, Arkansas

## Abstract

**Purpose and Objectives:**

The Centers for Disease Control and Prevention’s Sodium Reduction in Communities Program aims to reduce dietary sodium intake through policy, systems, and environmental approaches. The objective of our study was to evaluate changes in sodium levels over 5 years (2016–2021) in food served in school lunches as an outcome of a Sodium Reduction in Communities program in Arkansas’s largest school district.

**Intervention Approach:**

We collaborated with Springdale Public Schools (SPS) to reduce dietary sodium intake in school lunches through increased implementation of 1) food service guidelines, 2) procurement practices, 3) food preparation practices, and 4) environmental strategies. These activities were maintained from year 1 through year 5. Implementation priorities were informed each year by evaluation findings from the preceding year.

**Evaluation Methods:**

We collected lunch service records and information on nutritional content of menu items for the 30 schools under the direction of SPS’s Child Nutrition Department. We used a pretest–posttest quantitative evaluation design to analyze annual changes in the sodium content of meals, from baseline through year 5.

**Results:**

From baseline through year 1, SPS reduced sodium served per diner, per entrée offered, and per entrée served. These reductions were maintained from baseline through 5 years of follow-up. Mean sodium per 1,000 kcal per diner served was 1,740 mg at baseline and was lower in each of the 5 follow-up years: 1,488 mg (14% decrease) in year 1; 1,495 mg (14% decrease) in year 2; 1,612 mg (7% decrease) in year 3; 1,560 mg (10% decrease) in year 4; and 1,532 mg (12% decrease) in year 5. Energy served per diner remained stable.

**Implications for Public Health:**

Our study provides evidence for sustained sodium reduction strategies in a large ethnically and socioeconomically diverse school district, pointing to the potential benefit of implementing similar strategies in other school districts. The study also shows how program evaluation can be used to support sustainability.

**Summary**

**What is already known on this topic?**

Increased sodium intake in childhood is associated with cardiovascular characteristics that may lead to adult hypertension-related cardiovascular disease; however, approximately 75% of children exceed sodium intake guidelines.

**What is added by this report?**

We evaluated changes over 5 years in sodium amounts served in school lunches as an outcome of a Sodium Reduction in Communities program in Arkansas’s largest school district. The study contributes new evidence showing reductions in sodium were sustained from years 1 to 5.

**What are the implications for public health practice?**

Our study provides evidence for sustained sodium reductions in a large, diverse school district, pointing to the potential benefit of implementing similar strategies in other school districts and related settings.

## Introduction

Excess sodium intake is associated with hypertension and with cardiovascular disease (1–3), the leading cause of death in the US (4). Reducing excess sodium intake decreases hypertension (1,2) and is associated with decreased morbidity and mortality from cardiovascular disease (1–3). Increased sodium intake is associated with elevated blood pressure in childhood (5), which is associated with cardiovascular characteristics that may lead to adult hypertension-related cardiovascular disease (6). Reducing sodium intake can lower children’s blood pressure (6).

The 2020–2025 *Dietary Guidelines for Americans* identifies the daily recommended limit for sodium intake as 1,500 mg for people aged 4 through 8 years, 1,800 mg for people aged 9 through 13 years, and 2,300 mg for people aged 14 years or older (7). Children aged 1 through 18 in the US consume a mean of 2,905 mg sodium daily (8), and approximately 75% exceed the sodium intake guidelines (9). Strategies are needed to reduce children’s excess sodium intake (10).

The Centers for Disease Control and Prevention (CDC) initiated the Sodium Reduction in Communities Program (SRCP) in 2010 to reduce sodium intake in the US through policy, systems, and environmental approaches to improve access to reduced-sodium food products (11). Sites where SRCP has been implemented support sodium reduction strategies in food service venues that serve large populations, such as worksites, hospitals, schools, and universities. Each SRCP site evaluates the effectiveness of sodium reduction strategies in its venues.

The University of Arkansas for Medical Sciences (UAMS) received a 5-year SRCP award in 2016 to support implementation of sodium reduction strategies in northwest Arkansas. UAMS selected schools as a venue because they serve children in northwestern Arkansas communities at heightened risk for hypertension, including Marshallese populations, Latino populations, and populations with low incomes and food insecurity (12–14). Prior to applying for an SRCP project, UAMS met with local Marshallese and Latino communities and local food system groups (ie, community groups, a culinary arts program, and food vendors) to clarify which communities might benefit most. These meetings identified potential venues (eg, schools) and local priorities (eg, increasing availability of reduced-sodium foods for low-income and food-insecure populations). The group engagement process has been discussed in previous publications (13,15).

Springdale Public Schools (SPS) was the first school district to implement the project’s sodium reduction strategies in the region and was the focus of this study. At baseline (2016–2017 school year), SPS included 29 schools under the direction of the Child Nutrition Department. At baseline, SPS’s enrollment was 21,527 students in kindergarten through grade 12, making it the second largest school district in Arkansas. In 2018–2019, SPS became the largest school district in Arkansas. Among all SPS students at baseline, 46% were Latino, 35% were White, and 12% were Native Hawaiian or Pacific Islander, most of whom were Marshallese. At baseline, 71% of SPS students were eligible to receive free or reduced-price meals (16).

## Purpose and Objectives

Our initial evaluation from baseline to year 1 follow-up was discussed in a previous publication and showed reductions in mean sodium content served per diner and per entrée (13). This study includes a second, third, fourth, and fifth year of follow-up to evaluate changes in sodium served over time (ie, to investigate the extent to which reductions were sustained in years 2, 3, 4, and 5 of the program). This study also evaluates changes in the energy content of foods served.

## Intervention Approach

At baseline (2016-2017), SPS set a goal to move toward early compliance with anticipated US Department of Agriculture (USDA) National School Lunch Program sodium limits (eg, ≤740 mg per lunch for grades 9–12). At that time, USDA scheduled implementation of these limits for 2022–2023; however, in 2018–2019 the agency postponed implementation, and limits have since been increased (limits of ≤1,280 mg per lunch for grades 9–12 are now scheduled to go into effect in July 2023) (17–19). In addition, SPS set a target in 2016–2017 to reduce the sodium content of lunch entrées offered to meet USDA Smart Snacks in Schools guidelines of 480 mg or less (20). Entrées were defined as foods meeting the USDA National School Lunch Program’s classification of “meats/meat alternates.” The meats or meat alternatives category of a school meal includes meat, poultry, cheese, yogurt, eggs, peanut butter, and other protein-rich foods served as the main dish (21).

As in many school districts, policy decisions at SPS are made at the district level to guide nutrition programs in each district school, taking into account grade-level differences in student preferences and USDA school meal nutrition standards (19). District-level decisions about food service guidelines, procurement practices, menu planning, recipes, and cafeteria environments are implemented at each school in the district. Menus vary somewhat by grade level: 5 different lunch menus are offered per day at elementary, middle, junior high, high school, and a public charter school. Because SPS has significant Hispanic/Latino and Marshallese student populations, it makes an effort to incorporate culturally relevant foods into its menus. For instance, street tacos and elote (Mexican street corn) are served regularly throughout the school because of their popularity among local Hispanic/Latino communities. Teriyaki chicken and lo mein are served regularly because of their popularity among local Marshallese communities, whose cuisine often incorporates many Asian cultural influences.

Our SRCP intervention began by supporting SPS in selecting district-level sodium strategies and activities and then supporting implementation of activities at the district level and at each school within the district. The intervention at SPS included 4 sodium reduction strategies: 1) food service guidelines that address sodium, 2) procurement practices to reduce sodium content in purchased items, 3) food preparation practices to reduce sodium content of meals and menu items, and 4) environmental strategies and behavioral economics approaches to reduce sodium intake (eg, designing “Healthy Food, Healthy Future, Healthy Lunch” serving line signage to highlight healthy low-sodium choices, such as the salad bar). Implementation priorities were informed each year by evaluation findings from the preceding year. Sodium reduction activities (Table 1) affected each school, although some activities varied by grade level (eg, taste test procedures were different for first grade vs high school students).

**Table 1:** Sodium Reduction Intervention Activities Implemented by Springdale Public Schools During the Sodium Reduction in Communities Program, Springdale, Arkansas, 2016–2021^a^

Intervention strategy	Activities to address each strategy
Food service guidelines/standards that include sodium	Implemented comprehensive food service guidelines that include sodium reduction standards and practices. Example: SPS added language to specify preference for “low sodium” and “lower sodium” foods to procurement contracts and bids.
Procurement practices to reduce sodium content in purchased items	Implemented standardized purchasing lists with reduced-sodium items. Example: SPS began purchasing no-salt-added tortilla chips and no-salt-added lunch meat. Conducted taste tests of newly available or newly procured reduced-sodium ingredients/foods for students and staff. Example: SPS students participated in taste tests at Brightwater: Center for the Study of Food to try healthier alternatives to popular items such as macaroni and cheese. SPS child nutrition staff taste tested a grain bar concept that highlighted fresh vegetables, reduced sodium sauces, and whole grains.
Food preparation practices to reduce sodium content of meals and/or menu items	Implemented policy to eliminate “free salting.” Example: SPS child nutrition staff eliminated practice of salting foods at the end of meal preparation. Implemented rinsing of canned vegetables to reduce sodium content. Example: SPS emphasized through staff training the importance of rinsing all canned vegetables.
Environmental strategies/behavioral economics approaches to reduce sodium intake	Placed posters featuring sodium reduction messages in food preparation areas. Example: UAMS designed and supplied posters with spice blends and tips on seasoning without salt to all school kitchens for the staff to refer to when preparing meals. Moved saltshakers from dining tables and implemented flavor stations. Example: SPS offered flavor stations that featured no-salt-added spice blends for students to use on their meals.

Abbreviations: SPS, Springdale Public Schools; UAMS, University of Arkansas for Medical Sciences.

a All strategies were implemented by 2017, year 1.

Child nutrition directors met with UAMS staff 8 to 12 times per year in all 5 years of the project. These directors, along with cafeteria managers and frontline staff, participated in annual hands-on training in all 5 years. UAMS staff conducted the trainings in collaboration with instructors from Brightwater: A Center for the Study of Food and from the University of Arkansas Human Nutrition and Hospitality Management program. Trainings demonstrated food preparation practices to lower sodium in meals (eg, teaching knife skills to prepare fresh produce and preparing reduced-sodium salad dressings and sauces from scratch). Trainings also provided opportunities for information sharing between members of the UAMS staff and the SPS child nutrition staff.

By the end of year 1, SPS implemented activities encompassing all 4 strategies. Each year, the UAMS staff supported the SPS child nutrition staff in developing a work plan to ensure sustained implementation of each of the strategies. For example, the child nutrition director and team prioritized adding low-sodium items to their bid and procurement orders (eg, low-sodium spaghetti sauce), adjusting recipes to reduce sodium content in particular items (eg, using no-salt-added canned tomatoes in salsas), and offering training that taught practical and innovative ways of preparing reduced-sodium meals throughout years 1 through 5. SPS sustained each sodium reduction intervention activity through year 5.

A key aspect of implementation of this intervention was processing cafeteria service records and nutrition data shared by the school district. At baseline and during year 1, SPS provided these data in PDF (portable document format), and UAMS staff manually entered the data into Microsoft Excel (Microsoft Corp) for analysis. As the relationship between UAMS and SPS solidified, the staff of the two worked together to implement a process to export cafeteria service records and nutrition data in Excel format, which precluded the need for manual data entry. In year 4, UAMS staff developed code in R (R Foundation for Statistical Computing) to scrape necessary data from the exported data files for easier data management and analysis. A second important aspect of implementation was school district operational changes resulting from the COVID-19 pandemic. For year 4, interactions between school district staff and UAMS staff were conducted primarily via online video. In year 4, school meals were prepared and delivered in various ways. Meals were boxed and available daily at schools for pickup for students during the initial school closures early in the pandemic. Once schools re-opened, the district maintained boxed meals for virtual students but also initiated hybrid in-school delivery methods, including in-classroom meal service, cafeteria service, prepackaged meals, and individually prepared and portioned meals. Despite these operational changes, intervention activities were similar to previous years, and to preserve comparability with findings from previous years, the evaluation approach was identical.

## Evaluation Methods

Lunch menu data were collected annually from 29 SPS schools at baseline and year 1. Years 2 through 5 included data from 30 SPS schools, adding data from any district school that began following the district’s standard meal pattern in year 2. Baseline data were collected over 2 weeks in December 2016, prior to implementation of the intervention. From 2017 through 2021, annual follow-up data were collected for 2 weeks each October to minimize any potential seasonal variability. School district staff provided the number of diners and number of each menu item served for each day of observation. For each menu item, the name, ingredients, serving sizes, and nutrition information were provided from the school district’s records maintained in PrimeroEdge (Cybersoft Technologies, Inc). As a result, missing data were minimal (approximately 1% of all food items were missing nutrition information). Sodium (in mg) and energy (in kcal) were obtained for each menu item. When nutrition information was not available in PrimeroEdge, the information was collected from product websites, prior years’ records, or comparable school food formulated items in the USDA’s FoodData Central database (22). Nutritional content across all 6 data collection periods was calculated by using Excel 2019 (Microsoft Corp). This evaluation was determined to be exempt by the UAMS Institutional Review Board (#206008).

Total sodium content was calculated for all food served, including sides and condiments, across the observation period for each year. Mean sodium served per diner was calculated by dividing the total sodium content of all food items served by the total number of diners served during the observation period for each year.

To evaluate potential unintended consequences of the sodium reduction strategies on energy content, the mean of energy served per diner was calculated across the observation period for each year. The mean was calculated by dividing the total energy content of all food items served by the total number of diners served during the observation period.

To evaluate changes in sodium served relative to changes in energy, the mean number of mg of sodium per 1,000 kcal served per diner was calculated across the observation period for each year. This number was calculated by multiplying the mean mg of sodium served per diner by the quotient of the mean energy served per diner divided by 1,000.

Sodium mg per 1000 kcal = (1000/mean energy, kcal) * mean sodium, mg

A similar approach was taken to evaluate changes in the entrées offered on the menu. Mean sodium per entrée offered, mean energy per entrée offered, and sodium per 1,000 kcal per entrée offered were calculated across the observation period for each year. The calculations for entrées offered focused on menu-level changes (ie, not weighted by the number of entrées actually served to diners), so data for each entrée were included only once during each observation period.

To evaluate changes in entrées served, mean sodium per entrée, mean energy per entrée, and sodium per 1,000 kcal per entrée were calculated across the observation period for each year. These calculations took into account the number of times each entrée was actually served to a diner, so these calculations were weighted by the total number of each entrée actually served during the observation period.

We quantified the number and proportion of unique entrées offered and entrées served that met USDA’s Smart Snack in Schools (20) entrée sodium guideline of 480 mg or less for each year of observation.

## Results

For baseline and follow-up years 1 through 5, we measured the mean number of diners per lunch service and the results of the sodium and energy analyses (Table 2). The mean sodium served per diner was 1,140 mg at baseline and was lower in each of the 5 follow-up years: 978 mg (14% decrease) in year 1; 1,018 mg (11% decrease) in year 2; 1,062 mg (7% decrease) in year 3; 1,050 mg (8% decrease) in year 4; and 1,053 mg (8% decrease) in year 5. To ensure that changes in sodium served were not due to decreases in energy served, we tracked sodium mg per 1,000 kcal served per diner. Across the evaluation period, mean energy served per diner ranged from 655 kcal (baseline) to 687 kcal (year 5). Mean sodium per 1,000 kcal per diner served was 1,740 mg at baseline and was lower in each follow-up year: 1,488 mg (14% change) in year 1; 1,495 mg (14% change) in year 2; 1,612 mg (7% change) in year 3; 1,560 mg (10% change) in year 4; and 532 mg (12% change) in year 5.

**Table 2:** Mean Diners, Energy, and Sodium from Baseline through Year 5 at a School District Participating in the Sodium Reduction in Communities Program, Arkansas, 2016–2021^a^

Variables	Baseline	Year 1	Year 2	Year 3	Year 4	Year 5
Diners per lunch service, n (SD)	16,103 (984)	17,309 (342)	17,249 (617)	17,510 (543)	12,420^b^ (345)	15,793 (515)
Sodium served per diner, mg	1,140^c^	978^c^	1,018^c^	1,062^c^	1,050^c^	1,053^c^
Energy served per diner, kcal	655^c^	657^c^	681^c^	659^c^	673^c^	687^c^
Sodium per 1,000 kcal served per diner, mg	1,740^c^	1,488^c^	1,495^c^	1,612^c^	1,560^c^	1,532^c^
Sodium per entrée offered, mg (SD)	709 (426)	614 (286)	620 (299)	633 (312)	631 (299)	630 (307)
Energy per entrée offered, kcal (SD)	314 (105)	326 (118)	333 (119)	340 (131)	350 (129)	356 (132)
Sodium per 1,000 kcal per entrée offered, mg	2,262^c^	1,881^c^	1,863^c^	1,863^c^	1,802^c^	1,767^c^
Sodium per entrée served, mg (SD)	668 (330)	587 (215)	573 (267)	609 (233)	535 (209)	580 (240)
Energy per entrée served, kcal (SD)	314 (93)	343 (100)	325 (99)	336 (84)	313 (126)	345 (134)
Sodium per 1,000 kcal per entrée served, mg	2,126^c^	1,711^c^	1,764^c^	1,815^c^	1,707^c^	1,681^c^

a Data were collected immediately before intervention implementation in December 2016 (baseline) and again each October from 2017 through 2021 (years 1–5). Baseline and follow-up data were collected for 2 consecutive weeks of meal service each year for 29 schools (baseline and year 1) or 30 schools (years 2–5) in the Springdale, Arkansas, school district. At baseline, none of the intervention activities had been implemented.

b Year 4 diners per lunch service decreased because of state and local education policy changes resulting from the COVID-19 pandemic (eg, increased options to attend classes online).

c Values without SDs are those for which the means were calculated directly by dividing the total sodium or energy content by the relevant denominator. For example, mean sodium served per diner was calculated by dividing the total sodium content of all food items served by the total number of diners served during the observation period for each year. Quantities of sodium mg per 1,000 kcal presented in this table are based on calculations using unrounded values and will differ from quantities derived from calculations using the rounded values presented in this table.

Per entrée offered, the mean sodium was 709 mg at baseline and was lower in each of the 5 follow-up years: 614 mg (13% decrease) in year 1; 620 mg (13% decrease) in year 2; 633 mg (11% decrease) in year 3; 631 mg (11% decrease) in year 4; and 630 mg 11% decrease) in year 5. Across the evaluation period, mean energy per entrée offered ranged between 314 kcal (baseline) and 356 kcal (year 5). Mean sodium per 1,000 kcal per entrée offered was 2,262 mg at baseline and was lower in each follow-up year: 1,881 mg (17% decrease) in year 1; 1,863 mg (18% decrease) in year 2; 1,863 mg (18% decrease) in year 3; 1,802 mg, (20% decrease) in year 4; and 1,767 mg (22% decrease) in year 5.

Per entrée served, the mean sodium was 668 mg at baseline and was lower in each of the 5 follow up years: 587 mg (12% decrease) in year 1; 573 mg (14% decrease) in year 2; 609 mg (9% decrease) in year 3; 535 mg (20% decrease) in year 4; and 580 mg 13% decrease) in year 5. Across the evaluation period, mean energy per entrée served ranged from 313 kcal (year 4) to 345 kcal (year 5). Mean sodium per 1,000 kcal per entrée served was 2,126 mg at baseline and was lower in each follow-up year: 1,711 mg (20% decrease) in year 1; 1,764 mg (17% decrease) in year 2; 1,815 mg (15% decrease) in year 3; 1,707 mg (20% decrease) in year 4; and 1,681 mg, (21% decrease) in year 5.

At baseline, 22% (24 of 107) of unique entrées offered met the USDA Smart Snacks guideline of 480 mg of sodium or less. The percentage of unique entrées offered that met this guideline increased in year 1 (32%; 37 of 116) and remained above baseline in year 2 (34%; 44 of 131), year 3 (32%; 41 of 129), year 4 (37%; 32 of 87), and year 5 (36%; 43 of 118).

At baseline, 21% (41,703 of 196,138) of entrées served met the USDA Smart Snacks guideline of 480 mg of sodium or less. The percentage of entrées served that met this guideline increased in year 1 (27%; 44,421 of 167,251) and remained above baseline in year 2 (31%; 54,440 of 173,983), year 3 (30%; 52,002 of 172,219), year 4 (48%; 58,202 of 122,137), and year 5 (37%; 57,997 of 155,793).

## Implications for Public Health

From baseline to year 1, results of the SRCP intervention in SPS schools indicate reduced sodium served per diner, per entrée offered, and per entrée served. These reductions were sustained from years 1 to 5. Analyses of sodium mg per 1,000 kcal indicate that these reductions cannot be attributed to reduction in the energy content of food served. SPS’s school lunch program served a mean of 1,053 mg of sodium per diner in year 5, which is an 87 mg reduction from baseline. The year 5 mean result is in line with USDA National School Lunch Program sodium targets per lunch scheduled to go into effect in July 2023 (ie, ≤1,280 mg per lunch for grades 9–12, ≤1,225 mg per lunch for grades 6–8, and ≤1,110 mg per lunch for grades K–5) (19).

The year 5 mean of 1,532 sodium mg per 1,000 kcal served per diner exceeds the chronic disease risk reduction levels for sodium indicated in *Dietary Guidelines for Americans* *2020-2025 *(ie, 1,800 mg of sodium per day aged 9–13 years; 2,300 mg sodium/day aged ≥14 years) (7). However, this year 5 mean is a reduction of 208 sodium mg per 1,000 kcal from baseline. Simulation studies suggest that sodium reductions of this magnitude among US adults would produce significant increases in national productivity and significant reductions in national medical costs (23,24).

An important finding from our study is that sodium reductions were maintained from baseline through 5 years of follow-up. Across all measures, the amounts of sodium served or offered decreased markedly from baseline to year 1. From year 1 through year 5, sodium levels continued to decrease or returned partway toward baseline levels. The initial sodium reductions were largely maintained despite school district staff turnover and despite shifts in local, state, and national school nutrition policies related to the COVID-19 pandemic in years 4 and 5. Other challenges encountered included changing National School Lunch Program guidelines for sodium, shifting availability of healthy food from vendors (some of which was due to the COVID-19 pandemic), and demands on staff time needed to prepare healthy foods like fresh fruits and vegetables. Successful maintenance was due to lasting policy, systems, and environment changes the school district implemented during years 1 and 2 of the intervention (eg, comprehensive food service guidelines that included sodium reduction standards and practices).

Program evaluation was also used to support sustainability. Annually in years 1 to 5, UAMS staff provided school district staff with evaluation findings identifying the prior year’s highest sodium items. School district staff then worked with UAMS staff to select high-sodium items to focus on during the next year of intervention. School district staff had limited time to devote to sodium reduction activities, so this approach focused on modifying menus, recipes, and food preparation strategies that were likely to significantly affect sodium intake.

Our study had limitations. Cafeteria service data were not easily sorted into grade-level categories to facilitate direct comparisons with grade-level USDA National School Lunch Program sodium targets, which are increasingly salient as school districts prepare for lower sodium limits to take effect in July 2023. A second limitation is that, because of limits on staff resources, we focused on sodium served and sodium offered rather than sodium consumed. The study did not incorporate consideration of food waste. However, our study’s findings add to a growing body of evidence pointing toward SRCP effectiveness in reducing sodium across venues and sustaining sodium reductions over time (13,25,26).

Our study provides evidence for the sustainability of sodium reduction strategies in a large, diverse school district, pointing to potential benefits of implementing similar strategies in other school districts and related settings. The study contributes new evidence showing reductions in sodium were sustained over 5 years. Future evaluations will determine the extent to which reductions are sustained after active implementation support has ended

## References

[1] Cook NR , Appel LJ , Whelton PK . Lower levels of sodium intake and reduced cardiovascular risk. Circulation 2014;129(9):981–9. 10.1161/CIRCULATIONAHA.113.006032 24415713 PMC4181831

[2] Cook NR , Appel LJ , Whelton PK . Sodium intake and all-cause mortality over 20 years in the Trials of Hypertension Prevention. J Am Coll Cardiol 2016;68(15):1609–17. 10.1016/j.jacc.2016.07.745 27712772 PMC5098805

[3] Grillo A , Salvi L , Coruzzi P , Salvi P , Parati G . Sodium intake and hypertension. Nutrients 2019;11(9):E1970. 10.3390/nu11091970 31438636 PMC6770596

[4] Ahmad FB , Anderson RN . The leading causes of death in the US for 2020. JAMA 2021;325(18):1829–30. 10.1001/jama.2021.5469 33787821 PMC8145781

[5] Overwyk KJ , Zhao L , Zhang Z , Wiltz JL , Dunford EK , Cogswell ME . Trends in blood pressure and usual dietary sodium intake among children and adolescents, National Health and Nutrition Examination Survey 2003 to 2016. Hypertension 2019;74(2):260–6. 10.1161/HYPERTENSIONAHA.118.12844 31230545 PMC6657340

[6] Appel LJ , Lichtenstein AH , Callahan EA , Sinaiko A , Van Horn L , Whitsel L . Reducing sodium intake in children: a public health investment. J Clin Hypertens (Greenwich) 2015;17(9):657–62. 10.1111/jch.12615 26346989 PMC5034752

[7] US Department of Agriculture, US Department of Health and Human Services. Dietary Guidelines for Americans, 2020-2025. 2020. Accessed December 12, 2021. https://www.dietaryguidelines.gov/

[8] Wallace TC , Cowan AE , Bailey RL . Current sodium intakes in the United States and the modelling of glutamate’s incorporation into select savory products. Nutrients 2019;11(11):E2691. 10.3390/nu11112691 31703311 PMC6893472

[9] Brouillard AM , Deych E , Canter C , Rich MW . Trends in sodium intake in children and adolescents in the US and the impact of US Department of Agriculture guidelines: NHANES 2003–2016. J Pediatr 2020;225:117–23. 10.1016/j.jpeds.2020.04.048 32600669

[10] Batisky DL . With a grain of salt: can we make a difference? J Pediatr 2020;225:9–10. 10.1016/j.jpeds.2020.06.006 32522529

[11] Centers for Disease Control and Prevention, US Department of Health and Human Services. History and Impact of the Sodium Reduction in Communities Program. Accessed September 28, 2021. https://www.cdc.gov/dhdsp/programs/about_srcp.htm

[12] Gregory CA , Coleman-Jensen A . Food insecurity, chronic disease, and health among working-age adults. 2017. Accessed September 28, 2021. https://www.ers.usda.gov/webdocs/publications/84467/err-235.pdf

[13] Long CR , Rowland B , Langston K , Faitak B , Sparks K , Rowe V , Reducing the intake of sodium in community settings: evaluation of year one activities in the Sodium Reduction in Communities Program, Arkansas, 2016–2017. Prev Chronic Dis 2018;15:E160. 10.5888/pcd15.180310 30576274 PMC6307830

[14] US Department of Health and Human Services, Centers for Disease Control and Prevention, National Center for Health Statistics. Summary Health Statistics: National Health Interview Survey, 2018. Accessed September 28, 2021. https://ftp.cdc.gov/pub/Health_Statistics/NCHS/NHIS/SHS/2018_SHS_Table_A-1.pdf

[15] McElfish PA , Kohler P , Smith C , Warmack S , Buron B , Hudson J , Community-driven research agenda to reduce health disparities. Clin Transl Sci 2015;8(6):690–5. 10.1111/cts.12350 26573096 PMC4703475

[16] Arkansas Department of Education. Statewide information system reports — ADE Data Center. Accessed December 12, 2021. 2021. https://adedata.arkansas.gov/statewide/Default.aspx

[17] Food and Nutrition Service (FNS), USDA. Nutrition standards in the National School Lunch and School Breakfast Programs. Final rule. Fed Regist 2012;77(17):4088–167. 22359796

[18] Food and Nutrition Services (FNS), USDA. Child nutrition programs: flexibilities for milk, whole grains, and sodium requirements. Final rule. Fed Regist 2018;83(238):63775–91. 30540150

[19] US Department of Agriculture. Child nutrition programs: transitional standards for milk, whole grains, and sodium. Fed Regist 2022;87(25):6984–7023.

[20] US Department of Agriculture Food and Nutrition Service. A guide to Smart Snacks In School. FNS-623. 2022. Accessed June 22, 2022. https://www.fns.usda.gov/tn/guide-smart-snacks-school

[21] US Department of Agriculture. Meats/meat alternates. Food buying guide for child nutrition programs. 2017. Accessed July 5, 2022. https://foodbuyingguide.fns.usda.gov/Content/TablesFBG/USDA_FBG_Section1_MeatsAndMeatAlternates.pdf

[22] US Department of Agriculture, Agricultural Research Service. FoodData Central. Accessed July 5, 2022. https://fdc.nal.usda.gov/

[23] Dall TM , Fulgoni VL 3d , Zhang Y , Reimers KJ , Packard PT , Astwood JD . Potential health benefits and medical cost savings from calorie, sodium, and saturated fat reductions in the American diet. Am J Health Promot 2009;23(6):412–22. 10.4278/ajhp.080930-QUAN-226 19601481

[24] Dall TM , Fulgoni VL 3d , Zhang Y , Reimers KJ , Packard PT , Astwood JD . Predicted national productivity implications of calorie and sodium reductions in the American diet. Am J Health Promot 2009;23(6):423–30. 10.4278/ajhp.081010-QUAN-227 19601482

[25] Jordan J , Hickner H , Whitehill J , Yarnoff B . CDC’s Sodium Reduction in Communities program: evaluating differential effects in food service settings, 2013-2016. Prev Chronic Dis 2020;17:E72. 10.5888/pcd17.190446 32730201 PMC7417026

[26] Long CR , Spear MJ , Bogulski CA , Rowland B , Langston K , Faitak B , Reducing Sodium Intake in Community Meals Programs: Evaluation of the Sodium Reduction in Communities Program, Arkansas, 2016-2019. Prev Chronic Dis 2021;18:E63. 10.5888/pcd18.210028 34166180 PMC8269740

